# Predictive Markers for Malignant Urothelial Transformation in Balkan Endemic Nephropathy: A Case–Control Study

**DOI:** 10.3390/cancers12102945

**Published:** 2020-10-13

**Authors:** Gordana Kocic, Jovan Hadzi-Djokic, Jovana Cukuranovic-Kokoris, Mihajlo Gajic, Andrej Veljkovic, Rade Cukuranović, Dragoslav Basic, Ivan Jovanovic, Andrija Smelcerovic

**Affiliations:** 1 Department of Biochemistry, Faculty of Medicine, University of Niš, 18000 Niš, Serbia; andrej.veljkovic@medfak.ni.ac.rs; 2 Serbian Academy of Sciences and Arts, 11000 Belgrade, Serbia; jovan.hadzi-djokic@sanu.ac.rs; 3 Department of Anatomy, Faculty of Medicine, University of Niš, 18000 Niš, Serbia; jovana.cukuranovic.kokoris@medfak.ni.ac.rs (J.C.-K.); rade.cukuranovic@medfak.ni.ac.rs (R.C.); ivan.jovanovic@medfak.ni.ac.rs (I.J.); 4 Department of Pharmacy, Faculty of Medicine, University of Niš, 18000 Niš, Serbia; mihajlo.gajic@medfak.ni.ac.rs; 5 Clinic of Nephrology, Clinical Center Niš, 18000 Niš, Serbia; 6 Department of Surgery and Anesthesiology and Reanimatology, Faculty of Medicine, University of Niš, 18000 Niš, Serbia; dragoslav.basic@medfak.ni.ac.rs; 7 Clinic of Urology, Clinical Center Niš, 18000 Niš, Serbia; 8 Department of Chemistry, Faculty of Medicine, University of Niš, 18000 Serbia; andrija.smelcerovic@medfak.ni.ac.rs

**Keywords:** Balkan endemic nephropathy (BEN), aristolochic acid nephropathy, upper urothelial cancer, proteomic tumor markers, survivin

## Abstract

**Simple Summary:**

Balkan endemic nephropathy (BEN) is chronic kidney disease caused by intoxication with *Aristolochia* plant. Apart from subtle decline of renal function that eventually results in kidney failure, the patients are at increased risk for urothelial carcinoma (UC) development. Based on the observed UC markers, the aim of this study was to examine urinary and plasma levels of some these markers in BEN patients without carcinoma, in order to potentially identify those with predictive value. Our study revealed either plasma or urinary survivin levels as a potential predictors of future malignant transformation of urothelium.

**Abstract:**

Balkan endemic nephropathy (BEN) is a chronic tubulointerstitial disease frequently accompanied by urothelial carcinoma (UC). In light of the increased UC incidence and the markers observed in BEN patients with developed UC, the aim of the current case–control study is to assess survivin, p53 protein, growth factors and receptors (VEGF, VEGFR1, IGF I, IGF-1R and IGFBP5), tumor marker (TF)/CD142, circulating soluble Fas receptor and neopterin, as potentially predictive markers for UC in patients with BEN (52 patients), compared to healthy, age-matched subjects (40). A threefold increase was registered in both circulating and urinary survivin level in BEN patients. Especially noticeable was the ratio of U survivin/U Cr level five times the ratio of BEN patients associated with standard renal markers in multivariate regression models. The concentrations of VEGF, VEGFR1, (TF)/CD142, (sFas) were not significantly different in BEN patients, while urinary/plasma level demonstrated a significant decrease for VEGF. The levels of IGF I, IGFBP5 and IGF-1R were significantly reduced in the urine of BEN patients. Plasma concentration of neopterin was significantly higher, while urinary neopterin value was significantly lower in BEN patients compared to healthy controls, which reflected a significantly lower urine/plasma ratio and low local predictive value. As BEN is a slow-progressing chronic kidney disease, early detection of survivin may be proposed as potential predictor for malignant alteration and screening tool in BEN patients without the diagnosis of UC.

## 1. Introduction

Balkan endemic nephropathy (BEN) is a chronic tubulointerstitial disease, which prevails endemically in specific regions of Serbia, Bosnia and Herzegovina, Croatia, Bulgaria and Romania. The disease is usually observed in the populations in the basins of major rivers, such as the Danube, the Sava, the Drina, the Kolubara and the Great Morava [1,2,3,4].

The incidence of the disease has varied in the Balkan region over the last 40 years, with a decreasing tendency around the South Morava river and in Bulgaria. The average annual BEN rate used to be about 300 per 100,000, but it has declined about three times (97 per 100,000) [5]. Similar results have been observed in a study in Bulgaria [6]. In the Kolubara river region, the incidence of BEN was found to decrease between 1980 and 2000; however, an increasing tendency has been reported, especially in the population of 65-year-olds and above [7].

BEN is characterized by a silent progression of tubulointerstitial nephritis, which leads to renal sclerosis and to a chronic or even terminal renal failure. It may lead to dialysis and transplantation. Urothelial carcinoma (UC) is reported in BEN patients with a 100 times higher incidence compared to other risk factors for the occurrence of UC [8,9,10]. BEN exerts a serious impact on the social and economic status of the population, which affects families and the quality of life, and very often impairs ability to work.

In the complex pathogenesis of BEN numerous etiological factors have been identified as potential causes [11]. BEN sometimes affects whole families, which does not exclude synergistic genetic and epigenetic predisposition for the disease [12,13]. Molecular epidemiological evidence has confirmed that exposure to aristolochic acid, a derivative of the plant *Aristolochia clematitis*, may be the true cause of BEN [14,15]. In addition, aristolochic acid has been confirmed to produce a similar disease in China, known as Chinese herb nephropathy (CHN), also caused by the poisoning with *Aristolochia* plant. A common etiological factor, which is AA, together with similarities in the clinical expression and pathologic lesions throughout different stages of BEN and CHN led De Broe [4] to conclude that both BEN and CHN are two clinical expressions of a single entity named Aristolochic acid nephropathy (AAN). The study by Grollman et al. [15] confirmed that aristolochic acid may form DNA adducts with a renal tissue predilection, primarily at the A:T/T:A transversion level, most frequently for tumor suppressor protein p53, leading to DNA structure alterations and the onset of a malignant phenotype. It is estimated than 100,000 people are at risk of developing BEN, while 100,000,000 people worldwide may develop AAN [16].

The diagnostic criteria for BEN were established more than 40 years ago [17]. The clinical biochemical diagnostic set for BEN consists of the patient data (place of living), reduced glomerular filtration rate (GFR), proteinuria below 1 g/24 h, microalbuminuria, urinary markers of tubular injury (β2-microglobulin) and renal atrophy and nephrosclerosis detected by ultrasound [18]. One of the most reliable diagnostic markers of BEN is β2-microglobulin, typical for tubular proteinuria [19]. Chronic renal failure, which occurs as the consequence of BEN, does not produce any specific symptoms, while symptoms of anemia and loss of appetite are generally present in most renal diseases [18,20].

The progression of BEN may follow a course towards two clinical entities: terminal renal failure, UC or combined [20]. The disorders associated with stimulated proliferation and/or deregulated programmed cell death are important in the process of carcinogenesis. The disorders at the cellular level may refer to altered expression of tumor-suppressor proteins and a reduced expression of proteins or enzymes involved in apoptosis [21,22]. Progressive transformation of normal epithelial cell via premalignant into a malignant epithelial cell phenotype is a cascade which often involves the action of tumor promoters and mitogens. Acting in both autocrine and paracrine manner, they have a profound impact on both the growth factor-producing cells and their immediate microenvironment [23,24].

With regard to the significant prediction of UC in BEN patients and the previous results concerning the immunohistochemical expression of proteomic tumor markers in UC tissue specimens of BEN patients [21,22,25], the primary aim of the current study is to assess a potential predictive significance of proteomic markers, such as survivin, p53 protein, angiogenetic growth factor (VEGF), VEGF receptor (VEGFR1), insulin-like growth factor (IGF I),IGF I receptor (IGF-1R), IGF-binding protein (IGFBP5), tumor marker (TF)/CD142, circulating soluble Fas (APO1) receptor and neopterin in plasma and urine of BEN patients with no signs of UC. It is followed by the analysis of their urinary /plasma ratio to find a potential local predictive influence and by the analysis of their urinary level by creatinine ratio to predict the staging/severity of a patient’s chronic kidney disease. In the end, a calculation of the multivariate regression models was conducted in order to predict the associations between dependent variables with factors of interest. To our knowledge, this is the first study concerned with urinary to plasma ratio of proteomic tumor markers which may serve as predictive parameters for carcinogenesis in BEN patients without diagnosed UC. The information obtained may suggest which proteomic analysis may be used to predict early mechanisms of potential initiation and progression of a malignant process in urothelial cells in BEN patients.

## 2. Results

Table 1 provides the baseline characteristics and the clinical variables of patients with BEN and control subjects. Plasma creatinine significantly increased, while urinary creatinine clearance significantly decreased in BEN patients. All parameters of proteinuria and albuminuria increased significantly, but the most reliable for tubulointerstitial disease was β2-microglobulin. The results of regression analysis show a significant correlation between the values of urinary protein/creatinine index and urinary albumin/creatinine index as interdependent variables.

A threefold increase was registered for both circulating and urinary survivin level (Table 2). Especially noticeable was the five times increased ratio of U survivin/U Cr level in BEN patients, bearing in mind the association of surviving level with the severity of the disease. This may have a significant predictive value for a possible development of UC in BEN patients with no previously diagnosed UC.

No significant decrease of p53 was observed in plasma and urine of BEN patients. A twofold increase of p53 U/P ratio, together with the increased U p53/U Cr ratio, may have a local predictive value, but to a far lower extent than survivin (Table 2).

Plasma concentration of neopterin was significantly higher in patients with BEN, but urinary neopterin value in BEN patients was significantly lower than in healthy controls. This reflected a considerably lower urine/plasma ratio, providing an insignificant local predictive value (Table 2).

The concentration of plasma and urinary VEGF, VEGFR1, their U/P ratio, and urinary concentration to creatinine clearance ratio are shown in Table 2. There were no statistically significant differences in their concentrations. The U/P ratio indicated a significant decrease in VEGF, reflecting a low local predictive value.

The levels of insulin-like growth factor I (IGF I), insulin-like growth factor-binding protein 5 (IGFBP5) and IGF I receptor (IGF-1R) in the plasma and urine in BEN patients and controls are shown in Table 2. The levels of IGF I, IGFBP5 and IGF-1R were significantly reduced in urine of BEN patients. It should be emphasized that the ratio of U/P of IGF-1R was almost doubled in BEN. IGFBP5 was reduced in the plasma and urine of BEN patients; the ratio of its urinary to plasma level did not change significantly in BEN patients. The insignificant change in the urinary concentration to creatinine clearance excludes these markers as predictors associated with the severity of the disease.

The level of (TF)/CD142 and soluble Fas receptor (sFas) in the plasma and urine, as well as their U/P ratio did not change significantly (Table 2).

Multivariate regression models using enter method were performed to assess the associations between dependent variables. The values of the regression coefficients and their 95% CI were calculated. The associations statistically significant with standard renal markers are shown in Table 3. The level of plasma and urinary survivin, p53, VEGF, sFas and (TF)/CD142 were considered in significant associations with standard renal markers: urinary creatinine, creatinine clearance, urinary protein/creatinine ratio, urinary albumin/creatinine ratio. Urinary VEGF, plasma and urinary (TF)/CD142 showed a significant interrelation between Hb level and female gender, while plasma p53 and plasma VEGF showed significant interrelation with the patients’ age.

## 3. Discussion

The monitoring of the low molecular weight proteinuria and β2-microglobulin, reveals altered proximal tubules function for small molecule endocytosis, a primary lesion in BEN [19,26]. Obtained results confirmed urinary β2-microglobulin and α1-Microglobulin as “the gold standards” for diagnosis of BEN. In comparison to the nonspecific markers such as urinary protein level, urinary albumin level, urinary protein to creatinine index (UPCI), urinary albumin to creatinine index (UACI), they increased significantly. Our study was concerned with β2-microglobulin as a specific and sensitive urinary proteomic marker of tubular injury in BEN.

Since the changes in systolic and diastolic blood pressure were within the normal range of patients with BEN, the controls were chosen with regard to the age, gender, systolic and diastolic blood pressure level. Other authors have so far reported no changes in systolic and diastolic blood pressure level in BEN patients as well [27].

The results presented here suggest that the concentration of survivin is significantly higher in the plasma and urine of BEN patients. Previous articles concerning the expression of surviving in UC tissue specimens of BEN patients reported increased immunohistochemical expression of survivin as the most prominent marker of cancer of urothelial cells in BEN [22,25,26]. By exploring the Kaplan–Meier survival curves it was documented that increased survivin expression has a significantly shorter time for disease progression and development of metastases when compared to patients with normal survivin expression [28]. Standard laboratory diagnostics and endoscopy documented that urine cytology, cystoscopy and hematuria are the gold standards in the diagnostic assessment of patients with a high grade UC. At the same time, they noted the limitations, such as low sensitivity to detect carcinoma in situ and the appearance of a false-positive results in some other clinical states [29].

Survivin is known as baculovirus inhibitor of apoptosis, i.e., tumor-associated antigen (TAA) [30] and has been shown to inhibit apoptosis by the specific binding to caspase 3 and 7 [31]. It possesses the H3 histone-binding site, mediating in this way DNA stability [32]. In our previous study, the acetylation of specific sites of H3 and total H4 histones isolated from urothelial cells of patients with BEN was documented, as an important epigenetic chromatin modification in BEN [33]. Binding of survivin to H3 histones may alter the DNA stability. It is often referred to as a protein “at the border of life and death“. It has been proved an important marker for discriminating normal from neoplastic cells for theranostic purposes as chemotherapeutic target protein [33,34]. Survivin could be targeted by a complex theranostic platforms [35] or simpler theranostic agents, such as molecular beacons [36]. The research of survivin localization in the renal tissue has shown that it is located specifically on the apical membrane of proximal tubules, from where it is taken over via specific receptors, megalin or cubilin by proximal tubular cells [37]. The results obtained in our study have suggested that either plasma or urinary survivin may serve as a potential marker to predict future appearance of urothelial malignant transformation in BEN patients.

In our study, p53 protein concentration did not significantly change in plasma and urine specimens. However, the ratio of urinary to plasma p53 increased significantly, by almost 60% in BEN. Considering that GFR is significantly lower in BEN, such a ratio suggests that p53 may originate from urothelial cells. Tumor suppressor protein p53 is directly responsible for tissue apoptosis, called a “checkpoint” for cell cycle arrest, genetic stability maintenance, DNA reparation and inhibition of angiogenesis [38]. It usually exists as 12 isoforms because of alternative splicing [39]. Urothelial tumors associated with poor prognosis have demonstrated a broad spectrum of different mutations, responsible for the onset of malignant phenotype. It is almost impossible to pinpoint the exact one among thousands of potential mutations. It has been shown that mutated p53 protein has the tendency to accumulate, because of altered degradation, while non-mutated, “wild”-type is characterized by a short half-life [40]. Its accumulation is proportional to the tumor stage and correlates with the prognosis of urothelial carcinoma. It is especially important in suspect borderline cases or initial disease cases, particularly in those with dysplasia or transition into aggressive disease. In progressive urothelial cancer, elevated p53 qualified it as a valid marker of tumor progression and metastatic potential [40,41]. The data obtained in our study may point to a potential prediction of p53 for alterations at the local–urothelial level in BEN. The hypothesis is supported by the fact that a specific p53 mutation in the proximal renal tubular cells in experimental animals induces a decrease in the concentration of apoptotic proteins and reduced apoptosis [42]. The p53 knockout mice showed the resistance to renal tissue damage in experimental ischemic and toxic renal injuries [42,43]. The experimental nephrotoxic effect of aristolochic acid have shown significantly elevated p53 gene expression and p53 protein concentration [44].

Compared to control specimens, the concentration of soluble Fas receptor in the plasma and urine specimens of BEN patients did not significantly change. The extrinsic pathway of apoptosis starts with Fas ligand binding to transmembrane receptor Fas/APO-1. The Fas receptor is a membrane protein which belongs to the family of tumor necrosis factor-α (TNFα) receptors. Ligand-binding to Fas receptor triggers signaling cascade which ultimately results in DNA fragmentation [45]. Increased soluble Fas concentrations have been confirmed in membranoproliferative glomerulonephritis, correlating with its histological grade [46], in malignant melanoma and uterine tumors in which they correlated with corresponding tumor stages [47].

The concentrations of plasma neopterin was significantly higher in patients with BEN. In the urine, neopterin in BEN patients was significantly lower compared to the healthy individuals. The reason for increased neopterin value in the circulation of BEN patients may lie in the increased synthesis and/or reduced urinary elimination (reduced clearance). Neopterin is an organic compound containing nine atoms of carbon, with a molar mass of 253,215 Da, created as a breakdown product of GTP [48]. Neopterin is synthesized by macrophageal cells exposed to interferon gamma (IFNγ) [49]. Furthermore, neopterin initiates a complex cascade of cytokine activation, involving tumor necrosis factor-α (TNF-α) and interleukins, with consequential production of free radicals by activated inflammatory cells, which inevitably leads to tissue destruction [50]. Neopterin concentration is associated with chronic inflammatory conditions relevant for the pathogenesis of atherosclerosis, cardiovascular complications, but also in the development of end-stage renal disease, especially in kidney transplant patients, in whom inflammation can cause transplant rejection [50,51,52,53]. Considering the neopterin stability, compared to cytokines, its assessment may be useful for the renal function monitoring and the stage of inflammation monitoring [54]. A reduced clearance of this proinflammatory molecule may induce systemic effects. This may result in accelerated atherosclerosis, an impaired renal function and the development of coronary disease in the situation of chronic inflammation [55].

Vascular endothelial growth factor is a pro-angiogenic molecule and an important prognostic marker in UC. Our previous analyses concerned with the expression of both angiogenic growth factors and their receptors revealed that only VEGFR1 could be a significant discriminating factor between cancers of urothelial cells in BEN and non-BEN cancers [25]. The expression and concentration of VEGF and its receptor (VEGFR) in BEN patients was not significantly different from the controls (Table 2). Such a finding suggests that the downstream regulation is preserved, while lower concentrations of growth factors induce increased receptor expression. A preserved downstream regulation may be a typical feature of normal tissue, but not of carcinogenesis. Hypoxia may induce vasculogenesis, especially prominent in carcinogenesis. VEGFs regulate both vasculogenesis and angiogenesis [56,57]. Angiogenesis is a dynamic process in a tumor, vitally important for tumor cell survival and autonomy since it protects the tissue from hypoxia. Transcription factors induced by hypoxia (HIF-1, -2 and -3) may induce VEGF, angiopoietin 1 and 2, platelet-derived growth factor (PDGF) and placental growth factor expression and secretion, as our previous results observed [25,58]. The receptors for VEGF (VEGFR-1‚ VEGFR-2 and VEGFR-3) constitute a similarly heterogeneous family [59]. An increased synthesis of VEGF and its receptor has been documented in the UC forms observed in the presence of previously diagnosed BEN [60]. Their concentration strongly correlates with cancer invasiveness.

The IGF–IGFR axis is characterized by a very high oncogenic potential. Its oncogenic potential has been confirmed in prostate cancer, colorectal cancer and breast cancers [61,62]. The concentration of IGF I, its soluble receptor (IGF-1R) and IGFBP5 in plasma and urine are presented in Table 2. The concentration of IGF I and IGFBP5 were reduced, while urinary level of IGF-1R increased in urine, making the ratio of urinary to serum soluble growth factor almost identical. On the other hand, the soluble IGF-1R receptor increased in the urine, and the ratio of urinary and serum IGF-1R was almost doubled in BEN. Insulin-like growth factor (IGF I) was present in relatively high concentrations in the plasma, although it was mostly bound to its binding protein [62,63]. IGF I favors cellular expansion and promotes oncogenesis, leading to in situ carcinomas surrounding the basal membrane. Tumor suppressor genes and proteases complement the process and activate cellular invasion. IGF binds for two receptors—IGF-1R and IGF-2R. It also has a family of six binding proteins (IGFBP) reducing its bio-reactivity [64]. A genetic mutation that leads to the loss of receptor for IGF in experimental animals almost completely protects against the action of a number of studied oncogenic substances. This information shows that reduced concentrations of this binding protein may increase the IGF bioavailability.

Among the new potential tumor markers is a tissue procoagulant factor (TF)/CD142 (coagulation factor III/thromboplastin). No significant increase in BEN patients was documented in our study (Table 2). This type-I transmembrane glycoprotein represents a cell surface receptor and cofactor for blood coagulation factors VII and VIIa, responsible for thrombin and fibrin generation [59]. Having a significant impact on cell proliferation and migration (acting on PI3K and MAPK downstream signaling), CD142 becomes a new tumor promoter marker, responsible for tumor growth, angiogenesis and metastasis [65,66]. An increased expression was documented in urine of patients with bladder cancer, prostate cancer, pancreatic cancer, colorectal cancer, breast cancer, lung cancer and solid sarcomas and proposed as a new theranostic marker [64,66,67,68] According to the results of multivariate regression analysis (Table 3), its plasma and urinary level were documented in significant interrelation with Hb level in BEN patients.

## 4. Materials and Methods

In terms of patient and control subject recruitment, the study was designed as a case–control-type, as this is an appropriate strategy that permits the studying of rare diseases with long latency for manifestation, such as BEN (typical for endemic region). It was conducted from June 2016 to July 2018. Patients were admitted to the Institute of Nephrology, Clinical Center University Nis and BEN was diagnosed in patients according to standard diagnostic protocols [18]. The study was approved by the Ethical Committee of Faculty of Medicine (No. 01-1822/18). All patients recruited for the study signed the informed consent for the participation in the study. Enrolled patients (31 men and 21 women) were the native residents of the affected villages in a region endemic for BEN, with farming as a job occupation (around the river South Morava). Neither of BEN patients dropped out during a clinical study. Patients met all diagnostic criteria for enrolment into the study: familiar history of BEN, endemic place of living, reduced glomerular filtration rate (GFR), low-molecular weight proteinuria, microalbuminuria, urinary markers of tubular injury (β2-microglobulin), renal atrophy and nephrosclerosis detected by ultrasound [18,19]. Patient familiar history of the diseases and the history of the endemic place of living prior to onset of the disease were recorded via interview. Among the specific eligibility criteria for the enrolment were: the absence of any acute or chronic comorbidities, including cancer, the history of cardiovascular and other kidney diseases, systemic or immunologic conditions and inflammatory diseases. The control group comprised of 40 healthy examinees (23 men and 17 women), which met the same eligibility criteria: they were age-, gender- and job occupation-matched, the inhabitants of corresponding rural non-endemic regions for BEN, far about 40 km from endemic region, without acute or chronic comorbidities, including the history of cardiovascular and other kidney diseases, cancer, systemic or immunologic conditions and inflammatory diseases. Their health status was followed in the same way as that of the group of BEN patients.

For data collection, morning blood and urine samples from BEN patients and control subjects were taken, centrifuged at 3.000 rpm and kept at −80 °C. Blood samples were analyzed for hemoglobin (Hb) level, while standard plasma and urine biochemical parameters were measured by automated analyzer A24 for in vitro diagnosis (Biosystems SA).

Methods: Determinations of survivin, VEGF, VEGFR1, IGF I, IGFBP5, IGF-1R were performed using the standard ELISA assay (Quantikine R&D Systems, Minneapolis, MN, USA). Concentrations of p53, soluble Fas receptor (sFas) and (TF)/CD142 were measured using the standard ELISA assays (Abcam), while neopterin was determined by using ELISA assay (Biocompare, San Francisco, CA, USA). The sensitivity and the detection ranges of these assays were as follows: survivin (9.96 pg/mL; 31.2–2000 pg/mL); p53 (65 pg/mL; 0.23 ng/mL–15 ng/mL); sFAS (<3 pg/mL; 31–2 pg/mL–2000 pg/mL); (TF)/CD142 (20 pg/mL; 12.5 pg/mL–100 pg/mL); neopterin (<9.375 nmoL/L; 15.625–1000 nmoL/L); VEGF (9 pg/mL; 15.6–1000 pg/mL); VEGFR1 (13.3 pg/mL; 31.3–2000 pg/mL); IGF I (15 pg/mL; 31.2–2000 pg/mL); IGFBP5 (<10 pg/mL; 156 pg/mL–10,000 pg/mL); IGF1 R (250.00–16,000 pg/mL).

Data were analyzed using the SPSS statistical software for Windows Version 18.0 (Microsoft, Redmond, WA, USA) and expressed as median (5th–95th percentile) or number (percentage), as appropriate. To compare values of continuous variables between the two groups Mann–Whitney′s U test was performed. Pearson’s chi-squared test was used to compare categorical variables between the groups. Multivariate regression models using enter method were performed to estimate the associations between the dependent variables with factors of interest. A *p* value <0.05 was considered statistically significant.

## 5. Conclusions

The novelty of our study is that it reveals either plasma or urinary survivin, among the ten tumor markers documented to be expressed in UC of BEN patients, as a potential predictors of future malignant transformation of urothelium. This represents an entirely new mechanism of UC prediction that can be used long before the testing of a “gold standard” for UC. Therefore, these unique changes may indicate survivin as a potential prognostic tool and predictor for malignant alteration in BEN patients without diagnosis of UC. Although our results are preliminary, they may represent a basis for further research that should facilitate the potential screening of survivin level in a large number of BEN patients.

## Figures and Tables

**Table 1:** Baseline characteristics of Balkan endemic nephropathy (BEN) study cases and controls.

Parameters	BEN	Control	*p*
Male	31 (59.6%)	23 (57.5%)	0.866
Female	21 (40.4%)	17 (42.5%)	
Age (year)	73 (53.60–86.70)	73 (65.05–83.95)	0.377
Plasma Cr umol/L	123.20 (70.22–609.40)	85.20 (68.63–130.15)	0.000
Hb g/L	11.1 (8.7–141.6)	13.4 (12.71–157.9)	0.045
Glucose mmol/L	4.86 (3.80–6.60)	5.10 (4.31–6.69)	0.022
Creatinine clearance (CCr) mL/min	38.61 (7.93–89.41)	64.90 (23.09–106.70)	0.000
U Cr mmol/L	8.03 (1.77–23.79)	10.28 (5.20–23.57)	0.020
Urinary protein to creatinine index UPCI mg/mmol	21.91 (5.43–418.47)	11.72 (5.22–26.24)	0.000
Urinary albumin to creatinine index UACI mg/mmol	1.73 (0.18–61.72)	0.97 (0.20–12.17)	0.043
Urinary protein mg/L	225.0 (36.5–1517.5)	110.0 (50.0–499.0)	0.005
Urinary albumin mg/L	17.28 (1.44–415.24)	8.37 (2.14–232.24)	0.037
Beta2 microglobulin ug/L	99.81 (5.85–4794.00)	-	-

Values expressed as means (or percent) and median value (5th–95th percentile). *p* < 0.05 indicates statistical significance.

**Table 2:** Parameters serving as potential tumor markers in plasma and urine in patients with BEN.

Parameters	BEN	Control	*p*
Plasma survivin pg/mL	190.00 (29.67–1046.00)	66.67 (1.00–254.00)	0.002
U survivin	100.00 (53.33–354.67)	36.67 (0.00–894.67)	0.049
U survivin/U Cr	15.58 (2.54–29.87)	3.30 (0.00–41.07)	0.018
U survivin/P survivin	0.52 (0.34–0.76)	0.55 (0.29–0.66)	0.423
Plasma p53 ng/mL	0.17 (0.07–0.71)	0.24 (0.06–2.17)	0.241
U p53	0.13 (0.04–1.90)	0.11 (0.05–0.31)	0.804
U p53/U Cr	0.016 (0.050–0.900)	0.010 (0.008–0.050)	0.047
U p53/P p53	0.764 (0.091–0.953)	0.428 (0.221–0.501)	0.017
Plasma sFas pg/mL	3971.00 (1113.07–14,842.61)	4900.85 (684.92–14,231.42)	0.545
U sFas	351.31 (174.54–1465.95)	363.97 (72.88–1487.88)	0.465
U sFas/U Cr	36.07 (8.10–224.91)	32.14 (4.84–158.96)	0.380
U sFas/P sFas	0.088 (0.050–0.102)	0.074 (0.001–0.124)	0.405
Plasma neopterin nmol/mL	199.21 (7.59–312.46)	100.11 (10.75–246.57)	0.006
U neopterin nmol/mL	51.89 (15.7–249.37)	100.77 (7.75–294.03)	0.001
U neopterin/U Cr	11.47 (1.35–291.24)	118.53 (4.01–188.38)	0.021
U neopterin/P neopterin	0.260 (0.050–1.268)	1.000 (0.217–3.395)	0.001
Plasma VEGF pg/mL	344.57 (62.07–839.28)	365.29 (69.64–755.28)	0.395
U VEGF	31.00 (2,72–264.28)	43.86 (5.29–151.71)	0.219
U VEGF/U Cr	2.61 (0.17–50.08)	4.65 (0.52–11.71)	0.306
U VEGF/P VEGF	0.089 (0.043–0.123)	0.120 (0.044–0.134)	0.049
Plasma VEGFR1 pg/mL	144.67 (34.94–472.33)	110.78 (85.11–147.23)	0.125
U VEGFR1	51.89 (30.00–93.67)	43.00 (1.75–155.89)	0.114
U VEGFR1/U Cr	4.98 (1.28–13.81)	3.68 (0.19–21.73)	0.093
U VEGFR1/P VEGFR1	0.35 (0.14–0.67)	0.39 (0.21–0.57)	0.129
Plasma IGF-I pg/mL	188.40 (57.49–794.25)	326.80 (53.00–716.06)	0.645
U IGF-I	81.70 (31.10–159.15)	135.05 (61.00–200.66)	0.001
U IGF-I /U Cr	7.60 (2.15–20.60)	12.40 (5.20–33.31)	0.011
U IGF-I/P IGF-I	0.43 (0.09–0.81)	0.41 (0.05–0.90)	0.854
Plasma IGFBP5	558.50 (426.00–738.85)	625.00 (619.00–677.80)	0.044
U IGFBP5 pg/mL	582.00 (184.50–1154.00)	746.50 (251.00–1253.10)	0.027
U IGFBP5/U Cr	61.50 (17.10–127.95)	64.70 (21.50–126.03)	0.233
U IGFBP5/P IGFBP5	1.040 (0.043–0.158)	1.190 (0.035–0.159)	0.784
Plasma IGF-1R pg/mL	311.50 (1.00–854.50)	512.50 (1.00–735.70)	0.393
U IGF-1R	643.00 (302.50–1273.50)	551.50 (148.00–741.00)	0.009
U IGF-1R/C Cr	80.07 (24.10–117.02)	54.3 (31.50–100.03)	0.010
U IGF-1R/P IGF-1R	2.06 (0.17–12.08)	1.07 (0.11–6.08)	0.013
Plasma (TF)/CD142 pg/mL	103.59 (4.00–1260.11)	100.19 (5.55–336.06)	0.822
U (TF)/CD142	54.91 (12.00–379.14)	37.17 (3.77–138.30)	0.380
U (TF)/CD142 /U Cr	4.53 (0.17–24.42)	3.02 (0.54–16.84)	0.242
U (TF)/CD142 /P (TF)/CD142	0.53 (0.27–0.78)	0.37 (0.24–0.62)	0.058

Values expressed as means (or percent) and median value (5th–95th percentile). *p* < 0.05 indicates statistical significance.

**Table 3:** The interdependence of predictor variables in a multivariate regression model found statistically significant.

Variables	Multivariant Regression Analysis			
	B	95% CI		*p*
		Lower Bound	Upper Bound	
Plasma survivin and BEN patients	325.81	44.64	606.99	0.024
U survivin and CCr mL/min	6.39	0.88	11.91	0.029
U survivin and U Cr mmol/L	−12.21	−22.84	−1.58	0.030
U survivin and U albumin mg/L	-3.17	-6.29	−0.05	0.048
Plasma p53 and age	−0.002	−0.003	−0.001	0.006
Plasma p53 and P Cr umol/L	−0.013	−0.022	−0.005	0.006
Plasma p53 and CCr mL/min	−0.013	−0.022	−0.005	0.006
U p53 and UPCI mg/mmol	−0.065	−0.102	−0.028	0.005
U p53 and U Protein mg/L	0.007	0.002	0.012	0.013
U p53/P p53 and U albumin mg/L	0.001	0.0005	0.002	0.007
U p53/P p53 and UPCI mg/mmol	−0.010	−0.015	−0.005	0.003
U p53/P p53 and UACI mg/mmol	−0.010	−0.015	−0.005	0.003
U P53/P p53 and U protein mg/L	0.0010	0.0003	0.0020	0.007
P VEGF and female	−192.26	−358.92	−25.59	0.025
P VEGF and age	−17.72	−34.71	−0.72	0.041
U VEGF and Hb g/L	0.77	0.07	1.48	0.034
U VEGF and U protein mg/L	1.03	0.14	1.92	0.025
U VEGF and female	−95.91	−167.52	−24.29	0.016
U VEGF and Hb g/L	0.96	0.31	1.61	0.010
P VEGFR and Cr umol/L	0.70	0.24	1.16	0.004
P VEGFR and U Cr umol/L	0.81	0.06	1.56	0.036
Plasma CD 142 and BEN	30.90	0.72	61.09	0.045
Plasma CD 142 and Hb g/L	−0.78	−1.52	−0.04	0.040
U (TF)/CD 142 Hb g/L	0.96	0.31	1.61	0.010
U sFas /U Cr and P Cr umol/L	0.12	0.02	0.22	0.019
U sFas /U Cr and U Cr mmol/L	−1.80	−3.17	−0.44	0.010
U sFas and U Cr mmol/L	−2.41	−4.80	−0.02	0.048

Data analyzed using statistical software SPSS for Windows Version 18.0 (Microsoft, Redmond, WA, USA) and expressed as median (5th–95th percentile) or number (percentage), as appropriate. Mann–Whitney′s U test used to compare values of continuous variables between two groups. Pearson’s chi-squared test used to compare categorical variables between groups. Multivariate regression models using enter method used to estimate associations between dependent variables with factors of interest. *p* < 0.05 indicates statistical significance
